# Quality of clinical aspects of call handling at Dutch out of hours centres: cross sectional national study

**DOI:** 10.1136/bmj.a1264

**Published:** 2008-09-12

**Authors:** Hay P Derkx, Jan-Joost E Rethans, Arno M Muijtjens, Bas H Maiburg, Ron Winkens, Harrie G van Rooij, J André Knottnerus

**Affiliations:** 1 Department of General Practice, Maastricht University, Maastricht, Netherlands; 2 Skillslab, Maastricht University; 3 Department of Educational Development and Research, Maastricht University; 4 Department of General Practitioner Vocational Training, Maastricht University; 5 Centre for Quality of Care Research, Department of General Practice, Maastricht University; 6 Primary Care Out-of-hours Centre, Tilburg, Netherlands

## Abstract

**Objective** To assess the quality of telephone triage by following the consecutive phases of its care process and the quality of the clinical questions asked about the patient’s clinical condition, of the triage outcome, of the content of the home management advice, and of the safety net advice given at out of hours centres.

**Design** Cross sectional national study using telephone incognito standardised patients.

**Setting** The Netherlands.

**Participants** 17 out of hours centres.

**Main outcome measures** Percentages of clinical obligatory questions asked and items within home management and safety net advice, both in relation to pre-agreed standards, and of care advice given in relation to the required care advice.

**Results** The telephone incognito standardised patients presented seven clinical cases three times each over a period of 12 months, making a total of 357 calls. The mean percentage of obligatory questions asked compared with the standard was 21%. Answers to questions about the clinical condition were not always correctly evaluated from a clinical viewpoint, either by triagists or by general practitioners. The quality of information on home management and safety net advice varied, but it was consistently poor for all cases and for all out of hours centres. Triagists achieved the appropriate triage outcome in 58% of calls.

**Conclusion** In determining the outcome of the care process, triagists often reached a conclusion after asking a minimal number of questions. By analysing the quality of different phases within the process of telephone triage, evaluation of whether an appropriate triage outcome has been arrived at by means of good clinical reasoning or by an educated guess is possible. In terms of enhancing the overall clinical safety of telephone triage, apart from obtaining an appropriate clinical history, adequate home management and safety net advice must also be given.

## Introduction

Telephone triage can be described as the care process by which the degree of urgency of a patient’s clinical problem presented by telephone and the care needed are determined.^1^ This care process can be divided into the first phase of information gathering followed by the second phase of determining the degree of urgency and the care needed. The call handler, also called a triagist, actually handles the request for medical advice. In most cases this person is a specially trained nurse or a physician.^2^ The quality of telephone triage depends on the clinical knowledge and the communication skills of the triagist and his or her expertise in evaluating the information gathered.^3^

The safety of the advice given by triagists is often questioned,^4^ ^5^ ^6^ and most studies on the quality of telephone triage have focused on analysing the outcomes of triage.^7^ ^8^ ^9^ ^10^ ^11^ ^12^ ^13^ However, to assess the safety of telephone triage with more accuracy the quality of the care process itself needs to be analysed. This includes assessment of the clinical quality of questions asked as well as evaluation of the answers and the care advice given. For instance, if the triage outcome is assessed in isolation, the possibility of achieving a clinically acceptable outcome by chance may not be detected. Conversely, a clinically acceptable triage outcome might not occur owing to incorrect interpretation of the information gathered,^14^ even though the consultation process itself was carried out correctly. When the patient does not receive appropriate information on home management or safety net advice, inappropriate self care and delay in seeking medical care might occur.^15^

We could not identify any studies that investigated the safety of telephone triage by assessing the quality of each phase of the process of telephone triage. Therefore, the objective of this study was to make a global assessment of quality, specifically the quality of the clinical questions asked, the evaluation of the answers, the triage outcomes, and the content of the home management and safety net advice, in telephone consultations carried out by triagists at out of hours centres in the Netherlands.

## Methods

To assess the quality of clinical case handling at out of hours centres, telephone incognito standardised patients presented seven different clinical cases three times each to 17 different out of hours centres over a period of 12 months, totalling 357 calls.

### Cases, protocols, and scenarios

We applied several criteria for the selection of cases. The cases were to be based on symptoms that are known to present frequently on the telephone to out of hours centres,^7^ ^16^ and the care advice given as an outcome of the telephone consultation needed to be straightforward. In the Netherlands the triagist can select different triage outcomes depending on the degree of urgency: immediate and very urgent care (to be seen within one to two hours), urgent care (within two to six hours), routine care (care by a general practitioner the next day), or self care advice. For this study we decided for practical reasons that no case would need an outcome of immediate care or a home visit. The cases were designed to show the importance of accurate history taking, so that we could show whether the correct outcome had been determined by asking the required questions. Therefore, six of the seven cases to be presented consisted of three sets of two almost identical cases. For each of these parallel pairs of cases only one answer to an obligatory question differed (we called this the discriminating answer).

The box shows the obligatory questions for each case. Table 1 shows the scenarios for cases 6 and 7 to be played by the telephone incognito standardised patients, with the discriminating answers. Table 2 describes the discriminating answers for cases 2 and 3 and cases 4 and 5.

**Obligatory questions to be asked per case**

**Case 1: 5 year old child with fever**

- Can you describe the child’s behaviour now?
- How high is the temperature of the child?
- Has the child had a fit?
- Does the child have pain anywhere?
- Has the child got, or has he or she had, a headache?
- Can the child touch forehead on knees (or kiss knees)?
- Does the child seem breathless or is there indrawing of the chest/tummy?
- How much fluid has been taken in the past 12 hours?
- When did the child last pass urine?
- Does the child have a rash?
- Did the child recently travel abroad?

**Cases 2 and 3: adult with nosebleed**

- Duration of nosebleed?
- How many nosebleeds have you had in the past 48 hours?
- How much blood has been lost?
- Have you vomited any blood?
- Has there been a blow to the head?
- Are you feeling faint?
- Might you have a foreign object in the nose?
- Do you have bruises anywhere?
- Do you have a bleeding disorder?
- Do you take anticoagulant (blood thinning) treatment?

**Cases 4 and 5: adult with fever**

- Did the fever start with shaking/rigor?
- How high is the temperature?
- Do you have a stiff neck?
- Do you have any difficulties with breathing?
- Is there any pain when passing urine?
- How much fluid has been taken in the past 12 hours?
- When did you last pass urine?
- Any major long term health problem?
- Did you recently travel abroad?

**Cases 6 and 7: 5 year old child with vomiting**

- Can you describe the child’s behaviour now?
- How often did the child vomit in the past six hours?
- How much did the child vomit?
- Did the child vomit blood?
- Did the child drink in the past six hours?
- When did the child pass urine the last time?
- Did the child complain about pain when passing urine?
- Does the child have a rash anywhere?
- Does the child have fever?
- Does the child complain about a headache?
- Can the child touch forehead on knees (or kiss knees)?
- Does the child complain about abdominal pain?
- Does the child complain about photophobia?
- Did the child have a head injury recently?
- Did the child eat the wrong food?

**Table 1 tbl1:** Cases 6 and 7, with answers to questions to be given if asked. Opening by caller: “Good evening. I have a question. My son vomited a few minutes ago. I have some paracetamol. Should I give it to him?”

Questions	Answers to be given only when asked
Onset	Three hours ago (around 5 pm), at 6 30 pm, and a few minutes ago
Behaviour of child	Normal
Frequency of vomiting	Three times
Vomited blood	No
Diarrhoea	No
Abdominal pain	No
Dehydration:	
Drinking	Drank normally in past few hours
Micturition	One hour ago; no pain
Rash	No
Fever	No
Headache	Yes; complained of a slight headache
Earache	No
Stiff neck	No
Head injury	Case 6: No*
	Case 7: Yes*. Thank you for asking me. You have reminded me that this afternoon he fell while he was playing. He cried a lot. I also noticed a swelling at the back of his head
Wrong food	No
Medical history	No
Family	No; no one else is ill
Self care	None

*Discriminating answers for these two cases.

**Table 2 tbl2:** Discriminating answer for each parallel pair of cases

Case	Age	Clinical problem	Discriminating answer
1	5 years	Fever	Not applicable
2	Adult	Nosebleed	None
3	Adult	Nosebleed	Noticed several bruises in past few hours
4	Adult	Fever	None
5	Adult	Fever	Irregular usage of antimalarial drugs in past two weeks
6	5 years	Vomiting	None
7	5 years	Vomiting	Had head injury a few hours ago

To use up to date protocols, we decided to revise existing national protocols as these were four years old.^17^ When these were developed the Netherlands had little experience with out of hours services and with triagists (non-clinicians) handling all incoming calls. Protocols for telephone triage include not only questions about possible causes and the consequences of the symptom presented but also questions to compensate for the lack of visual information about a patient’s clinical condition.^18^ They also include advice about home management care to be given if the triage outcome does not include a face to face consultation (for example, type and dose of analgesic drug). Finally, they include advice about the circumstances in which the patient should call back (“safety net advice”).^19^ ^20^ ^21^

To reach consensus and achieve standardisation for this study we presented these protocols to a panel of general practitioners with experience in telephone triage at out of hours centres. They agreed on the obligatory questions that should be asked about the different clinical symptoms and what should be discussed with the patient in relation to home management and safety net advice.

### Telephone incognito standardised patients

Lay people have been used as “unannounced” or “incognito” standardised patients for many years to assess the performance of students and healthcare professionals during face to face and telephone consultations. Evidence shows that this method is a reliable and valid instrument to assess clinical performance.^22^ ^23^ ^24^ ^25^

Specially trained telephone incognito standardised patients presented the cases. They received a scenario with information on their personal data, the clinical problem, their personal situation (such as feeling anxious), and the answers to be given to the obligatory questions if they were asked. If they were invited to come for a consultation at the out of hours centre, they were instructed to ask for the reason for this request. Another panel of general practitioners validated the scenarios and considered them realistic and representative of cases that might present to out of hours centres. No additional obligatory questions were needed to determine clinical urgency. They agreed that it was reasonable for telephone incognito standardised patients to receive self care advice for cases 1, 2, 4, and 6 and to be advised to be seen the same evening for cases 3, 5, and 7.

To increase the chances of speaking to different triagists, the telephone incognito standardised patients called in the evening between 7 pm and 9 pm on different days of the week. They made the calls from their private home, pretending to be in the area of the involved out of hours centre. They audio recorded the calls for further assessment.

### Out of hours centres

In October 2004 105 out of hours centres were operative in the Netherlands. In 2005 they received a total number of 3.3 million calls. Of these, 38% resulted in self care advice (source: VHN; Dutch organisation of out of hours centres).

We asked all centres for permission to be selected for the research study. Of the 98 centres that gave permission, we selected 17 out of hours centres but did not inform them of this. We did a balanced selection, whereby for each of the 12 provinces of the Netherlands the size of the population in that province determined the number of out of hours centres to be selected. We then selected centres at random. After the study we asked the 17 out of hours centres by letter whether they had detected a telephone incognito standardised patient during the previous 12 months.

### Assessment and analysis

The required frequency of presentation of the cases developed was based on generalisability theory.^23^ ^26^ In a first batch, the seven cases were presented five times to five of the 17 selected out of hours centres. We assessed the quality of a centre on the basis of the average quality score over cases and repeated calls. In a generalisability analysis we estimated the variance components associated with centres, cases, centre-case interaction, and repeated calls (nested within a centre-case combination). We then used this information in a decision analysis to calculate the generalisability coefficient for varying numbers of repeated calls; the generalisability coefficient is equal to the ratio of the variance of interest, the variance of the quality score over centres, and the total variance, the sum of all variance components. With the number of repeated calls set to three, the generalisability coefficient was 0.9, a value that is considered sufficiently high even for high stakes examinations. Therefore, the remaining 12 out of hours centres were called three times for the same seven cases.

We made a transcript of every recorded call and assigned each call to two medical students as raters. They scored calls independently by using the standard protocol as a checklist. They rated each item by marking “Yes” (=1: question is asked/advice is given) or “No” (=0: question is not asked/advice is not given).

For each call, we recorded the following variables as indicators of quality: percentage of obligatory questions asked in relation to the agreed standard set of questions; percentage of items within home management and safety net advice in relation to the agreed standard set of items; percentage of obligatory questions asked in relation to all questions asked; percentage of appropriate care advice given in relation to the required care advice. For each of these variables we used the average percentage of the two raters for further analysis of each case for all out of hours centres and for each out of hours centre for all cases. We used the statistical software package SPSS version 15 for descriptive statistics and analysis of variance.

## Results

Tables 3 and 4 and the figure[Fig fig1] show the findings for cases and out of hours centres. For 58% of all calls the required urgency level advised was as set by the scenario panel. Urgency was underestimated in 41% of calls and overestimated in 1% of calls.

**Table 3 tbl3:** Results for each case for obligatory questions asked versus standard, home management and safety net advice provided versus standard, obligatory questions versus all questions asked, and appropriate outcome compared with standard (n=51 calls per case)

Case	Obligatory questions asked among total questions asked			Mean home management and safety net advice provided versus standard		Obligatory questions asked versus standard (%)	Appropriate care advice given versus required advice (%)
	No of questions in standard set	Mean (SD) %		No of items in standard set	Mean (SD) %		
1	11	30 (19)		9	36 (21)	47	100
2	10	19 (7)		8	56 (23)	54	96
3	10	21 (10)		8	53 (23)	68	2
4	9	16 (13)		9	26 (13)	36	94
5	9	17 (16)		9	26 (13)	32	22
6	15	24 (14)		5	41 (23)	70	96
7	15	19 (14)		5	43 (23)	73	2
Total		21 (14)			40 (23)	54	58

**Table 4 tbl4:** Results for each out of hours centre for obligatory questions asked versus standard, home management and safety net advice provided versus standard, obligatory questions versus all questions asked, and appropriate outcome compared with standard (n=21 calls per out of hours centre)

Out of hours centre	Mean (SD) % obligatory questions asked versus standard	Mean (SD) % home management and safety net advice provided versus standard	Mean % obligatory questions asked among total No of questions asked	Appropriate care advice given versus required advice (%)
1	16 (13)	37 (19)	52	43
2	15 (12)	36 (23)	50	57
3	18 (17)	36 (31)	51	48
4	19 (11)	34 (18)	57	48
5	42 (24)	44 (23)	59	52
6	15 (9)	40 (26)	51	57
7	23 (15)	43 (24)	58	62
8	28 (15)	35 (20)	57	67
9	21 (15)	36 (23)	53	52
10	19 (10)	46 (23)	46	57
11	22 (13)	48 (21)	65	57
12	21 (11)	44 (21)	59	52
13	16 (12)	34 (24)	55	57
14	17 (8)	51 (20)	50	52
15	23 (11)	45 (20)	55	54
16	17 (9)	35 (24)	47	52
17	22 (8)	43 (24)	59	57
Total	21 (14)	40 (23)	54	58

**Figure fig1:**
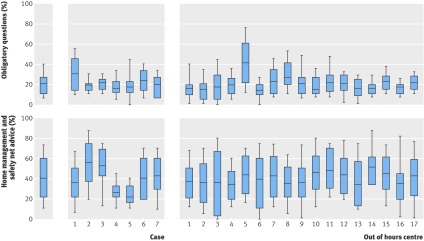
Box plots (distributions) of percentage obligatory questions asked versus standard and percentage home management and safety advice provided versus standard. Left panel shows distribution for all calls (n=357), middle panel shows distributions per case (51 calls per box plot), and right panel shows distributions per out of hours centre (21 calls per box plot). Each box plot shows mean value (solid line), 25th and 75th centiles (lower and higher boundaries of grey box) indicating central part, and 10th and the 90th centiles (grey “whiskers” outside box) indicating outer parts of distribution

The overall mean percentage of obligatory questions asked was equal to 21% of the standard. The mean percentage varied between 15% at out of hours centre number 6 and 42% at number 5, but at all centres the percentage was consistently far below the standard (100%). We found a similar pattern between cases: some variation between cases existed, but for all cases the mean percentage was far below the standard.

The overall mean for the content of home management and safety net advice was 40% (lower left panel of figure[Fig fig1]). For this variable the variation between cases was more pronounced, from 26% for the case of an adult with fever to 56% for the case of an adult with a nosebleed. However, for all cases and all centres the major finding was a mean percentage far below the standard.

Table 3 shows that of all questions asked, on average 54% were obligatory questions. This percentage varied from 32% to 73% between cases and from 46% to 65% between out of hours centres.

The figure[Fig fig1] shows the difference in performance between individual triagists at the same out of hours centre. Some of them asked many obligatory questions, whereas others asked none. Some managed a score of almost 90% for relevant home management and safety net advice, and others gave none at all.

The same triagist handled the same case twice in 3% of the calls; all other calls were handled by different triagists. The triagists referred 2% of the telephone incognito standardised patients to the out of hours centre in the region of their permanent residence without any triage. None of the 17 out of hours centres indicated that they had detected any call made by a telephone incognito standardised patient.

For 153 calls the required care advice resulting from the telephone triage was to come to the out of hours centre. This care advice was given on 17 occasions. On six occasions triagists suggested that the reason why the patient needed to be seen at the out of hours centre the same evening was the use of antimalarial treatment, and on four occasions it was because of travel to an African country. For seven urgent care outcomes no clinical reason could be identified.

General practitioners returned a call on six occasions to a telephone incognito standardised patient acting as an adult with fever (case 5) in which the “patient” mentioned the use of Malarone. The outcome of these return calls was self care advice on four occasions, and on two occasions the telephone incognito standardised patient was advised to be seen the same evening for further assessment (the reason given was: “We have to exclude malaria as the cause of your fever”).

## Discussion

The results of this study identify shortcomings and educational needs in telephone triage, indicated by assessing the quality of phase one (asking appropriate questions and evaluating the answers) and phase two (giving care advice) of the process of telephone triage. The mean score for obligatory questions asked was 21% of the agreed standard. Answers to obligatory questions were not always evaluated clinically correctly. The quality of the information given on home management and safety net advice was also consistently below the standard. The appropriate triage outcome was reached in only 58% of calls.

### Strength and weaknesses

Triagists are known to respond very accurately to cases of high urgency but not to moderate and low urgency cases such as those presented in this study.^27^ Our study shows underestimation of urgency in 41% of calls and overestimation of urgency in 1%, which is in line with other results.^12^ ^28^ ^29^ However, our design using sets of parallel cases with a discriminating answer shows that assessing only triage outcomes might lead to incorrect conclusions on the safety of telephone triage being drawn, even if the correct outcome is determined.

As none of the 17 out of hours centres had detected any of the telephone incognito standardised patients and different triagists handled 97% of all calls, we conclude that this study reflects the day to day performance of call handling at those centres.

### Cases, protocols, and scenarios

Some members of the scenario panel considered case 2 (adult with nosebleed) as likely to be a frequently presented case but case 3 (as case 2 but with bruises) as quite uncommon. We decided to present this case, as it is an example of an uncommon but potentially life threatening symptom,^30^ ^31^ which can be dealt with safely by adequate triage.

In our study many obligatory questions remained unasked, and triagists often asked questions that the protocol and scenario panels did not consider as required to determine an urgency level. This means that for telephone triage, history taking was often not carried out effectively. For instance, for the case of nosebleed triagists often asked the question: “Do you have an allergy or hypertension?” This shows that the triagist investigated a possible cause of nosebleed, which is not relevant for determining the degree of its urgency, whereas questions about bruises or the amount of blood loss were not asked.

Research shows that triagists often ask few questions and do not use or follow protocols.^32^ ^33^ ^34^ ^35^ ^36^ The safety of any care process is likely to be improved by following protocols, but little is known about what extent of improvement might occur. In our study the analysis of variance for obligatory questions asked compared with the standard showed that the overall mean significantly and substantially differed from 100%. Although agreed standards are seldom met, we conclude that the number of obligatory questions asked at the different out of hours centres was far too low to determine clinical urgency.

Triagists at out of hours centre number 5 asked significantly more obligatory questions for all cases than did those at any other centre. However, this did not result in a higher number of appropriate outcomes than at the other centres. Determining the reason for this would need further investigation.

An example of misinterpretation of an answer took place on a Friday evening when the triagist of an out of hours centre said to the telephone incognito standardised patient with nosebleed (case 3): “You’d better see your doctor next week because you said you have a bleeding nose and several bruises, so there might be something wrong with your blood.” Our findings on (mis)interpretation of answers by triagists and general practitioners confirm that in addition to asking the right questions, understanding the relevance of the answers is equally important.

Triagists gave a small amount of home management and safety net advice relevant to the nature of the clinical problem presented. Further research is needed to investigate whether triagists are aware of the importance of this advice, especially if self care advice is given.

### Conclusion

Triagists seemed to carry out a rapid clinical scan before they came to a conclusion, without considering in sufficient detail different causes for a symptom or its possible consequences. Even when the triagist gave the required self care advice, for nearly all calls this choice could be considered to be the correct choice only by means of an educated guess as so few obligatory questions were asked. This study also shows that triagists should ideally recommend a low urgency level of care only after thorough history taking. The quality of information on home management and safety net advice varied to a large extent and was consistently low for each case and each out of hours centre.

### Implications for further research

Telephone triage should aim at minimising risks to a patient’s health. The safety of telephone triage might be enhanced by using computer based decision support systems.^1^ ^37^ On the basis of the findings of this study, such a program should have as a minimum requirement protocols with advice on obligatory questions to be asked, recommendations for urgency outcome, and relevant home management and safety net advice. Little is known about the conditions in which these systems are to be used.^38^ ^39^ Accordingly, research is needed on the design and implementation of these systems to support telephone triage effectively and safely. Our results also indicate a need for future studies on training for and quality assurance of telephone communication. In addition, study of the documentation of telephone calls is needed.

**What is already known on this topic**

Research on the quality of telephone triage is often focused on the quality of the outcomes
Little is known about the quality of the different phases of the care process of telephone triage by triagists

**What this study adds**

Care advice was given after asking (too) few obligatory questions
Answers to those questions, if asked, were not always interpreted correctly by the triagist or general practitioner
The quality of home management and safety net advice was low

## Notes

We thank Ron Hoogenboom for helping with the statistical analysis of all data.

Ethical approval: Not needed.

Provenance and peer review: Not commissioned; externally peer reviewed.

## Notes

**Cite this as:** *BMJ* 2008;337:a1264
